# The Th17/Treg balance and the expression of related cytokines in Uygur cervical cancer patients

**DOI:** 10.1186/1746-1596-8-61

**Published:** 2013-04-15

**Authors:** Zhifang Chen, Jianbing Ding, Nannan Pang, Rong Du, Wei Meng, Yuejie Zhu, Yi Zhang, Cailing Ma, Yan Ding

**Affiliations:** 1 Gynecology Department, First Affiliated Hospital of Xinjiang Medical University, Xinjiang 830054, P. R. China; 2 Xiangya Hospital of Central South University, Changsha 410000, P. R. China; 3 Xinjiang Medical University, Department of Immunology, Xinjiang 830054, P. R. China

**Keywords:** Uygur, Cervical cancer, Treg, Th17, Cytokine

## Abstract

**Background:**

The fine balance of Th17/Treg is crucial for maintenance of immune homeostasis. The objective of this study was to investigate the balance of Th17/Treg and the expression of related cytokines in Uighur cervical cancer patients.

**Methods:**

Peripheral blood was collected from 65 cases of cervical cancer patients, 42 cases of cervical CIN patients and 40 healthy people. Flow cytometry was used to detect the percentages of T cell subsets, including CD3^+^ T cells, CD4^+^ T cells, CD8^+^ T cells, Treg cells and Th17 cells. ELISA assay was conducted to detect expression levels of TGF-β, IL-6, IL-10, IL-17, IL-23 and IFN-γ.

**Results:**

There were no significant difference in the levels of CD3^+^ T cells, CD4^+^ T cells, CD8^+^ T cells, and the ratio of CD4^+^/CD8^+^ among the cervical cancer group, the CIN group and the healthy control group. However, compared with the healthy control group, the percentages of CD4^+^ CD25^+^ Treg, CD4^+^CD25^+^CD127^-^ Treg, CD4^+^IL17^+^ Th17, CD4^+^CD25^+^Foxp3^+^, CD4^+^CD25^-^ Foxp3^+^, CD8^+^CD25^+^CD127^-^Treg and CD8^+^CD25^+^Foxp3 were significantly higher in the cervical cancer group and the CIN group. Similar results were also found in the Th17/Treg ratio and the related cytokines. There was no significant difference between the cervical cancer group and the CIN group. Additionally, Th17 cell levels were positively correlated with IL-6, IL-23 and IL-17. Also, Treg cell levels were positively correlated with TGF-β, IL-10 and IL-6. Contrarily, Treg cell levels and IFN-γ were negatively correlated.

**Conclusions:**

Our data indicated that the Th17/Treg balance was broken in peripheral blood of cervical cancer patients. Analysis of Th17/Treg balance may have a significant implication in diagnosing cervical cancer.

**Virtual slides:**

The virtual slide for this article can be found here: http://www.diagnosticpathology.diagnomx.eu/vs/1813823795931511

## Background

The morbidity rate and mortality rate of cervical cancer rank the second place in the female genital tract malignancies. Annually there are nearly 500,000 new cases worldwide and half of them die of cervical cancer [1]. Surgery and (or) radiotherapy are the most commonly used treatments for cervical cancer. The interventional chemotherapy is also an effective adjuvant therapy. However these methods are still limited. With the rapid development of tumor immunology and molecular biology, biological therapy has become an important measure for the treatment of malignant tumors. In Xinjiang Uygur women, the morbidity rate and mortality rate of cervical cancer were 459/100000-590/100000 and 15.78/100000, significantly higher than the other ethnic groups living in the same environment. The onset age of cervical cancer was also earlier than other ethnic minorities in the country. Furthermore the mortality rate of cervical cancer in Xinjiang Uygur women ranked the first place in the ethnic minorities of our country [2]. Thus it is urgent to study the Uygur cervical cancer-specific diagnostic method and therapy.

Cervical cancer is primarily caused by persistent infection with high-risk human papilloma-virus (HPV). HPV infection, in most cases, is self-limiting and can be eradicated by humoral and cell-mediated immune response. This suggests that immunoregulation may play an important role in cervical cancer carcinogenesis. However, there is limited information on Th17/Treg and their related cytokines in cervical cancer bearing hosts, especially in Uyghur women. Importantly, only a minority of the cases progress to cervical precancerous lesions. And the process from precancerous lesions to invasive cervical cancer takes about ten years. Therefore, it is of great importance to make effective screening of precursor lesion. The most widely applied screening methods are cytological examination and HPV test. Currently, new methods have been implicated. Liu et al. [3] applied the genomic amplification of the human telomerase gene (hTERC) as a supplementary method to screen cervical cancer in high-risk patients. For the detection of high-grade cervical intraepithelial neoplasia (CIN), Chen et al. [4] used the genomic amplification patterns of human telomerase RNA gene and C-MYC. Monoclonal antibody D2-40 against M2A antigen and p16 have also been used as immunoreactive makers to identify cervical cancer [5,6]. However, little is known about the diagnostic roles of immune cells and cytokines in cervical cancer.

Studies have shown that the CD4^+^ T cell subsets include helper T cells type 1 (Th1), helper T cells type 2 (Th2), CD4^+^ CD25^+^ regulatory T cells (Treg) and helper T cells 17 (Th17) [7,8]. Under different circumstances, CD4^+^ T cells can differentiate into these cell subsets and secret different cytokines to mediate immune response. For example, under the induction of IL-12 and interferon-γ (IFN-γ), CD4^+^ T cells can differentiate into Th1 cells, and produce IFN-γ. With the induction of IL-4, CD4^+^ T cells can differentiate into Th2 cells and secrete IL-4, IL-5 and IL-13. With the induction of transforming growth factor-β (TGF-β), CD4^+^ T cells can differentiate into Treg cells, which secrete TGF-β and express Forkhead family protein 3 (Foxp3). Under the induction of the TGF-β and IL-6, CD4^+^ T cells can differentiate into Th17 cells and produce IL-17, IL-21, IL-23 and other cytokines. As a negative regulator, IL-10 has a strong immunosuppressive effect and plays a critical role in reducing the immune damage. By secreting IL-10, Th2 cells can inhibit the function of Thl cells and regulate the balance of Th1/Th2. In addition, IL-10 can regulate autoimmune inflammatory damage such as EAE and contribute to the role of anti-inflammatory by Treg cells. In addition to Th2 and Treg cells, Th17 cells can also secrete a small amount of IL-10.

The discovery of Th17 cells derived from the study of the mechanisms of autoimmune diseases. Th17 cells mainly secrete IL-17 and play many roles in immune response. They can promote the proliferation and differentiation of a variety of cells. Also they are involved in the proliferation, maturation and chemotactic activity of neutrophils. Additionally they can co-stimulate the activation of T cells and promote the maturation of dendritic cells. Treg cells can negatively regulate the activation and proliferation of autoreactive T cells. Hence they are closely related to maintaining immune tolerance, preventing the occurrence of autoimmune diseases and inhibiting anti-graft rejection and tumor immunity. Previous studies reported that the immune responses against tumor were mainly mediated by Thl cells. If the Thl/Th2 ratio was imbalanced because of the Th2 cells increase, the anti-tumor immune response of the body would be severely weakened, leading to the malignant growth of the tumor. Current studies have found that Treg cells and Th17 cells also played an important role in anti-tumor immune response. Xiang et al. [9] reported that the levels of Th17 cells and Treg cells were imbalanced in inflammatory response and autoimmune diseases. However whether this imbalance is involved in the regulation of tumor immune is unknown and has aroused much attention. The relationship between the imbalance of Th17/Treg and tumor needs further investigation. In this study, we analyzed the percentages of Th17 cells, Treg cells and FoxP3 positive T cells. We also detected the expression levels of IL-6, IL-10, IL-17, IL-23, IFN-γ and TGF-β. Our results provide a further understanding of the differentiation status of immune cells in cervical cancer of Uygur women.

## Methods

### Subjects

Sixty-five cases of cervical cancer patients who did not undergo surgery and were enrolled in our hospital from January 2009 to October 2011 were selected as the Uygur cervical cancer group (UCC group). They were aged between 36 to 60 years, with a mean age of 45.50 ± 6.12 years. Forty-two cases of cervical cancer patients with intraepithelial lesions that did not undergo any treatment were selected as the CIN group. They were aged between 26 to 58 years, with an average age of 44.24 ± 5.67 years. Forty healthy volunteers who did physical examination in our hospital at August 2010 were selected as the healthy control group (control). They were aged between 24 to 66 years, with an average age of 45.35 ± 6.17 years. There were no significant differences in age among the three groups. None of the enrolled subjects had any of the following diseases: diabetes, hypertension, cardiovascular disease, pregnancy, acute or chronic infectious disease and a history of metastatic tumors. The clinical features of the subjects were summarized in Tables 1, 2 and 3. The histology and clinical stage of cervical cancer patients were classified based on 2000FIGO installments. Conduct of clinical trials for this study was approved by the Medical Ethical Committee of First Affiliated Hospital of Xinjiang Medical University. All participants signed the informed consent before sampling.

**Table 1:** The clinical data of the Uyghur cervical cancer patients

**Characteristic**	**Category**	**N = 65**
FIGO stage	Ia	12(18)
	Ib	14(21)
	IIa	34(52)
	IIb	5(7)
Histology type	SCC	56(86)
	ADC/ADSC	9(14)
Tumor differetiation	Well	11(17)
	Moderate	21(32)
	Poor	33(51)
Lymph node metastases	Positive	23(35)
	Negative	42(65)
Tumor size(cm)	< 4	31(48)
	≥ 4	34(52)
Infiltration depth(mm)	< 15	28(43)
	≥ 15	37(57)
Vasionvasion	Yes	21(32)
	No	41(63)
	Unknown	3(5)

Note: Pathological characteristics of cervical cancer in patients of the UCC group were shown.

**Table 2:** The clinical data of the cervical CIN patients

**No. of patient**	**42**
Age (years)	
Median	44
Range	26-58
Clinical stage	
CIN II	7
CIN III	35

Note: Pathological characteristics of cervical cancer in patients of the CIN group were shown.

**Table 3:** The clinical features of the three groups

**Clinical features**	**UCC group**	**CIN group**	**Control group**
	**(n = 65)**	**(n = 42)**	**(n = 40)**
Age (median age)	36-60 (45.50 ± 6.12) years	26-58 (44.24 ± 5.67) years	24-66 (45.35 ± 6.17) years
Smoking	7	5	4
Drinking alcohol	3	2	2
Diabetes	1	0	0
Vascular disease	2	0	3
Cancer of any other type	0	0	0
Acute infection	0	0	0
Chronic infection	0	2	0
Chemotherapy	1	0	0
Irradiation therapy	0	1	1
Pregnancy	0	0	0

Note: Direct comparison of the clinical features of the subjects enrolled in this study.

### Antibodies, reagents and instruments

Mouse anti-human antibody, fluorescein isothiocyanate (FITC)-CD25 antibody, phycoerythrin (PE)-CD127 antibody, phycoerythrin-cy5 (PE-cy5)-CD4 antibody, allophycocyanin (APC)-CD3 antibody, phycoerythrin-cy7 (PE-Cy7)-CD8 antibody, PE- Foxp3 antibody and the isotype control antibody were purchased from Beckman, USA. PE-labeled IL-17A monoclonal antibody (clone No. ebio64DEC17) was purchased from eBioscience, America.

ELISA kits for the detection of human TGF-β, IL-6, IL-10, IL-17, IL-23 and IFN-γ were obtained from the Bender MedSystems, Austria.

The flow cytometer was purchased from Becton Dickinson, USA.

### Sample preparation

Peripheral blood mononuclear cells (PBMCs) were obtained from peripheral blood by Ficoll-Hypaque density centrifugation (2500 r/min for 20 minutes at room temperature). PBMCs were re-suspended at a density of 2 × 10^6^ cells/ml in Roswell Park Memorial Institute (RPMI) media 1640 with GlutaMAX. The RPMI 1640 media were supplemented with 100 U/ml penicillin, 100 μg/ml streptomycin and 10% fetal calf serum (Gibco, USA).

### Flow cytometric analysis

For analysis of Th17 cells, the cell suspension was stimulated with 20 ng/ml phorbol 12-myristate-13-acetate and 1 μg/ml ionomycin in the presence of 2 mmol/ml monensin (Sigma-Aldrich, USA) in 24-well plates. After stimulating for 4 hours (37°C, 5% CO_2_), the cells were collected and washed once in phosphate buffered saline (PBS). The cells were then incubated with APC-CD3 and PE-Cy5-CD4 at 4°C for 30 minutes. After surface staining, the cells were fixed and permeabilized according to the manufacturer’s instruction, and then stained with anti-human PE-IL-17.

For analysis of Treg cells, the cell suspension was transferred into tubes and washed in PBS. Then the cells were stained with APC-CD3, PE-cy5 -CD4, PE-Cy7-CD8 and FITC-CD25 at 4°C for 30 minutes. Then some of the cells were stained with PE-CD127. The other cells were incubated with PE-Foxp3 after fixation and permeabilization according to the manufacturer’s instruction.

All stained cells were analyzed by flow cytometry (FACSCalibur) and FlowJo software (Tristar, USA). In the flow cytometry, the forward angle scattering light (FSC) and side scattering light (SSC) were adjusted to select the lymphocytes. Different cell subsets were detected by different cell labeling and gating. Cellquest software was used for data analysis and the percentage of positive cells was recorded.

### Enzyme-linked immunosorbent assay (ELISA)

Serum of all groups were collected and stored in −80°C before analysis. ELISA assay was performed according to the manufacture’s instructions of the ELISA kits (Bender MedSystems, Austria). The OD value at 450 nm was measured. The concentrations of TGF-β, and IL-6, IL-10, IL-17, IL-23 and IFN-γ were calculated according to the standard curve.

### Statistical analysis

The experimental data were expressed as mean ± s.e.m. and analyzed by SPSS17.0 software. The age difference among groups was compared using the t test. The difference between the groups was analyzed by variance analysis. Correlation analysis was performed with Spearman rank correlation analysis. A p value of less than 0.05 was considered statistically significant.

## Results

### Percentages of T cells and T cell subsets in the peripheral blood of the three groups show no significant difference

**Figure 1:**
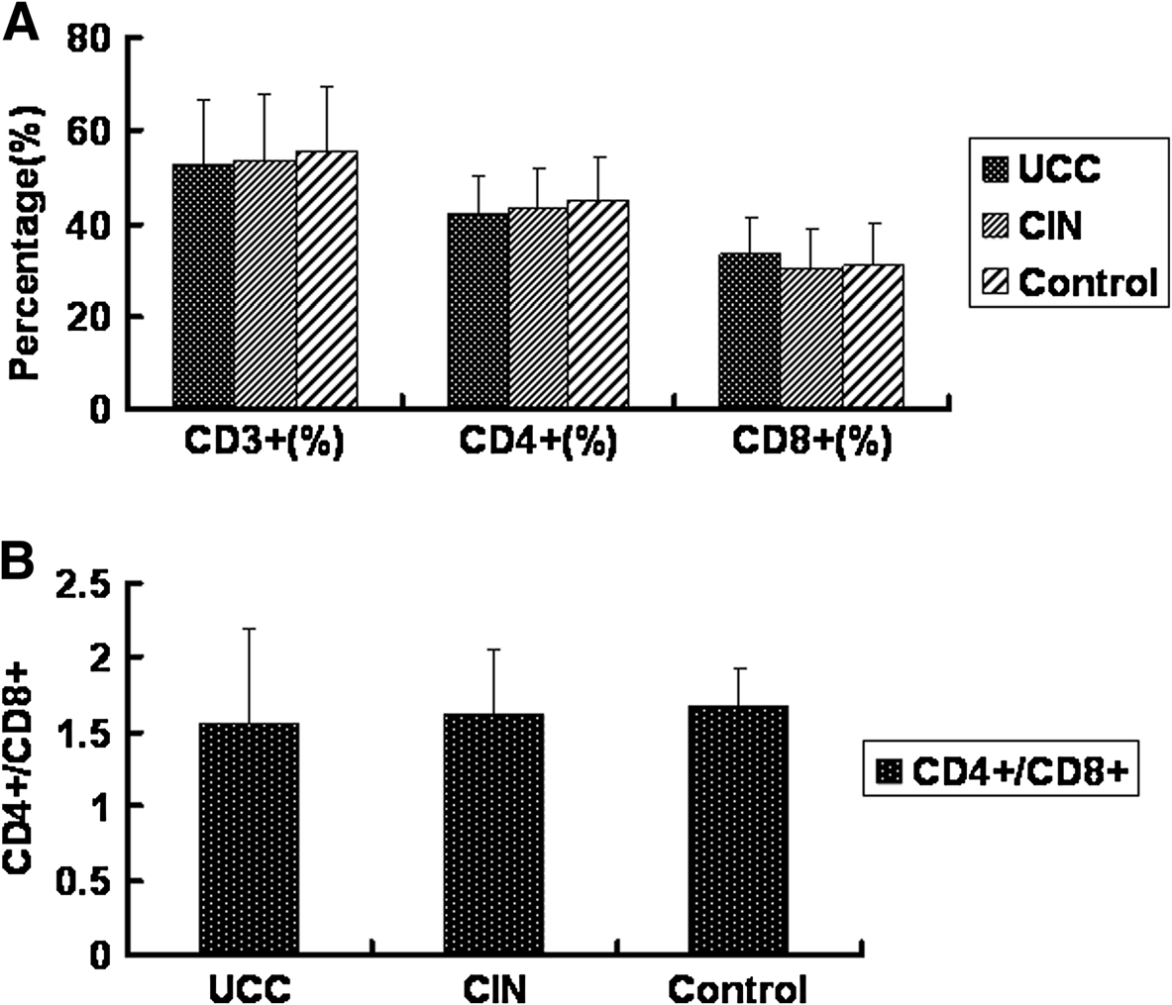
**Analysis of CD3**^**+ **^**T cells, CD4**^**+ **^**T cells, CD8**^**+ **^**T cells and the CD4**^**+**^**/CD8**^**+ **^**ratio in the peripheral blood of three groups.** Peripheral blood samples were collected from 65 cases of cervical cancer patients, 42 cases of cervical CIN patients and 40 cases of healthy people. Flow cytometry was used to conduct this experiment. (**A**) Percentages of CD3^+^ T cells, CD4^+^ T cells and CD8^+^ T cells; (**B**) The ratio of CD4^+^/CD8^+^ in the UCC group, the cervical CIN group and the healthy control group. p > 0.05.

The percentages of T cells and T cell subsets in the peripheral blood indicate the functional status of the immune system. To check the immune function of the three groups, we analyzed the levels of CD3^+^ T cells, CD4^+^ T cells and CD8^+^ T cells, and the CD4^+^/CD8^+^ ratio. The cells were analyzed by flow cytometry and the quantitative results were shown in Figure 1. The percentages of CD3^+^ T cells in the UCC group, the CIN group and the healthy control group were 52.80% ± 13.97%, 53.8% ± 14.07% and 55.56% ± 13.86%, respectively. The percentages of CD4^+^ T cells in the UCC group, the CIN group and the healthy control group were 42.10% ± 8.22%, 43.33% ± 8.68% and 45.07% ± 9.52%, respectively. The percentages of CD8^+^ T cells in the UCC group, the CIN group and the healthy control group were 33.18% ± 8.35%, 30.03% ± 9.14% and 30.95% ± 9.20%, respectively. Statistically, for all the T cell subsets (CD3^+^ T, CD4^+^ T and CD8^+^ T cells) detected, the differences among the three groups were not significant (p > 0.05). In addition, the ratio of CD4^+^/CD8^+^ in the UCC group, the CIN group and the healthy control group were 1.56 ± 0.64, 1.62 ± 0.43 and 1.67 ± 0.26, respectively. Also there was no significant difference among the three groups (p > 0.05). These results suggest that the immune function of the cervical cancer patients and the cervical CIN patients was similar to that of the healthy individuals.

### Percentages of Treg cells and Th17 cells are elevated in cervical cancer patients and CIN patients

**Figure 2:**
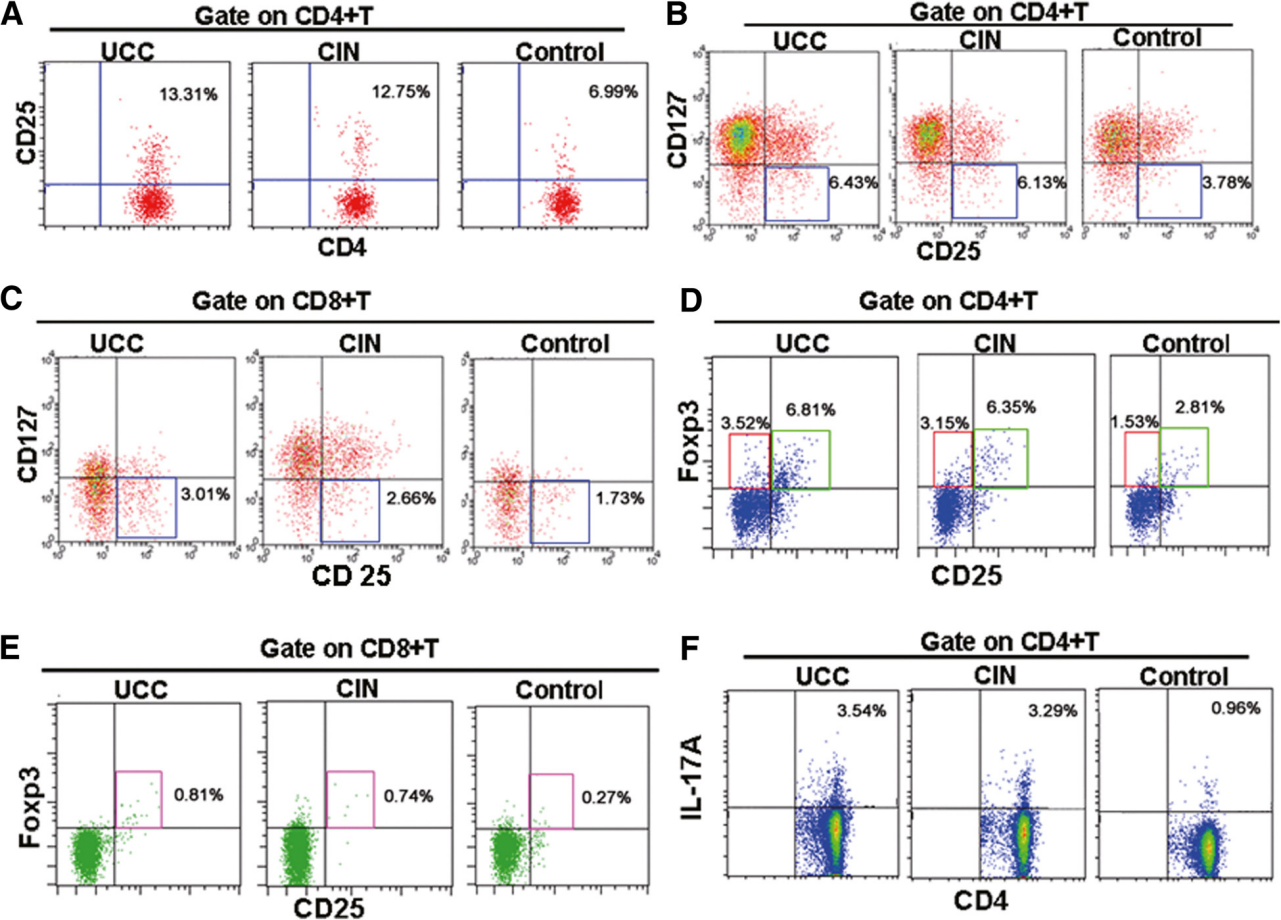
**Flow cytometric analysis of Treg cells and Th17 cells in the peripheral blood of three groups.** Peripheral blood samples were collected from 65 cases of cervical cancer patients, 42 cases of cervical CIN patients and 40 cases of healthy people. Representative flow cytometric results were shown. The indicated percentages in each figure represented the levels of the analyzed cell subsets. (**A**) Percentages of CD4^+^CD25^+^Treg cells in CD4^+^T cells; (**B**) Percentages of CD4^+^CD25^+^CD127^low^Treg cells in CD4^+^T cells; (**C**) Percentages of CD8^+^CD25^+^CD127^low^Treg cells in CD8^+^T cells; (**D**) Percentages of CD4^+^CD25^+^ FOXP3^+^Treg cells and CD4^+^CD25^-^FOXP3^+^Treg in CD4^+^T cells; (**E**) Percentages of CD8^+^CD25^+^ FOXP3^+^Treg cells in CD8^+^T cells; (**F**) Percentages of CD4^+^IL-17^+^Th17 cells in CD4^+^T cells.

**Figure 3:**
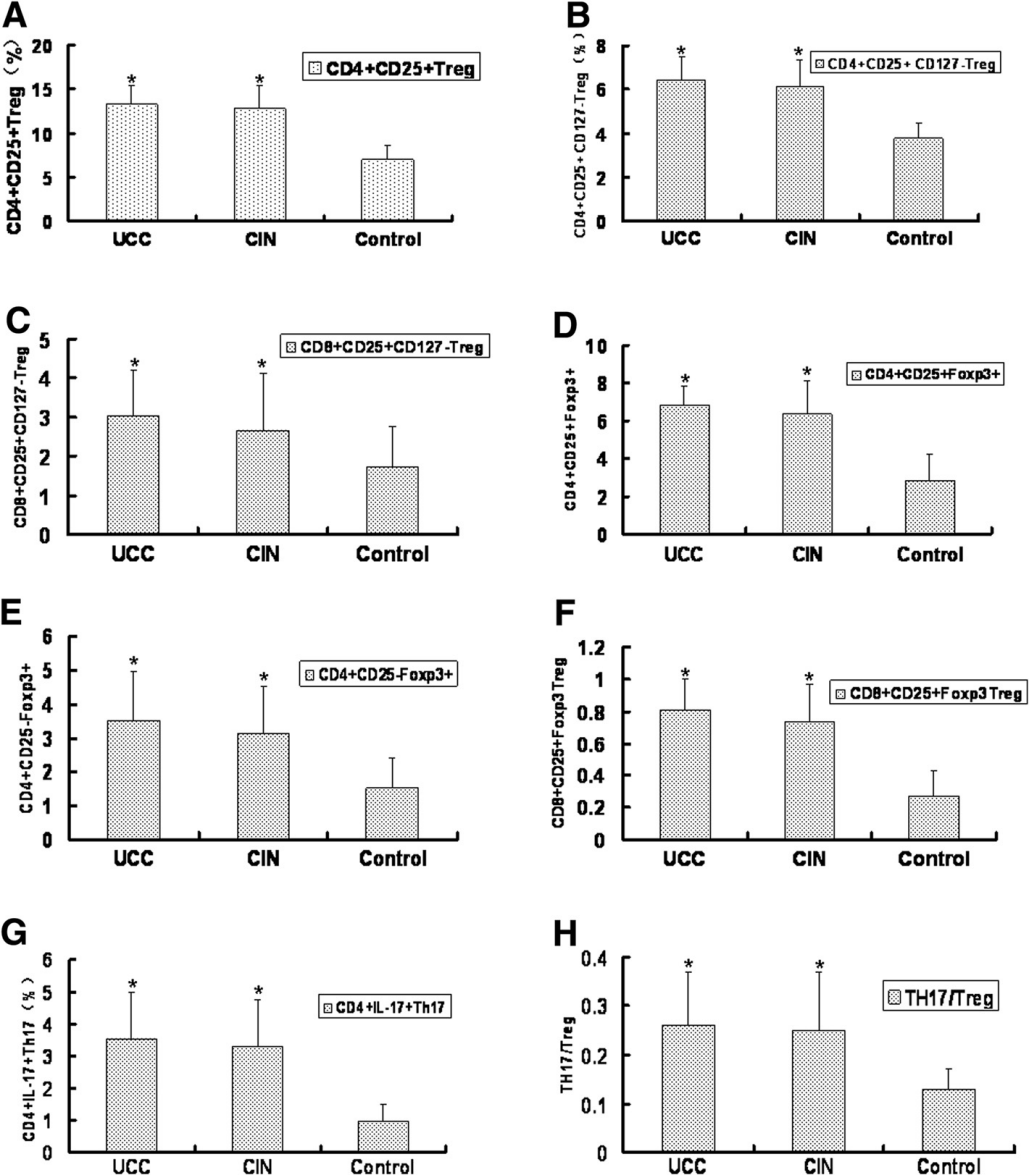
**Quantitative analysis of Treg cells and Th17 cells in the peripheral blood of three groups.** Peripheral blood samples were collected from 65 cases of cervical cancer patients, 42 cases of cervical CIN patients and 40 cases of healthy people. Quantitative results of the flow cytometric analysis were shown. (**A**) Percentages of CD4^+^CD25^+^Treg cells in CD4^+^T cells; (**B**) Percentages of CD4^+^CD25^+^CD127^low^Treg cells in CD4^+^T cells; (**C**) Percentages of CD8^+^CD25^+^CD127^low^Treg cells in CD8^+^T cells; (**D**) Percentages of CD4^+^CD25^+^ FOXP3^+^Treg cells in CD4^+^T cells; (**E**) Percentages of CD4^+^CD25^-^FOXP3^+^Treg in CD4^+^T cells; (**F**) Percentages of CD8^+^CD25^+^ FOXP3^+^Treg cells in CD8^+^T cells; (**G**) Percentages of CD4^+^IL-17^+^Th17 cells in CD4^+^T cells. (**H**) Ratio of Th17/ Treg in three groups. Each experiment was performed three times. Percentages of cells in the UCC group and the cervical group were compared with those in the healthy control group. *p < 0.05.

Percentages of Treg cells and Th17 cells in the peripheral blood of the three groups were analyzed by flow cytometry. Cells were labeled with different surface markers. The following subsets of CD4^+^CD25^+^Treg, CD4^+^CD25^+^CD127^-^Treg, CD8^+^CD25^+^CD127^-^Treg, CD4^+^CD25^+^Foxp3^+^, CD4^+^CD25^-^Foxp3^+^, CD8^+^CD25^+^Foxp3^+^ and CD4^+^IL17^+^Th17 cells were analyzed respectively. And the ratio of TH17/Treg was also checked. Representative flow cytometry results were shown in Figure 2 and the quantitative results were shown in Figure 3. As indicated in Figures 2A and 3A, the percentages of CD4^+^CD25^+^Treg cells in the UCC group and the cervical CIN group were 13.31% ± 2.02% and 12.75% ± 2.61%, statistically higher than those in the control group (6.99% ± 1.62%) (p < 0.05). There was no significant difference between the UCC group and the CIN group (p > 0.05). Similarly, the percentages of CD4^+^CD25^+^CD127^-^Treg cells (Figures 2B and 3B), CD8^+^CD25^+^CD127^-^Treg cells (Figures 2C and 3C), CD4^+^CD25^+^Foxp3^+^ cells (Figures 2D and 3D), CD4^+^CD25^-^Foxp3^+^ cells (Figures 2D and 3E), CD8^+^CD25^+^Foxp3^+^ cells (Figures 2E and 3F) and CD4^+^IL17^+^Th17 cells (Figures 2F and 3G) were also significantly higher in the UCC group and the CIN group compared with the control group (p < 0.05). No significant difference was found between the UCC group and the CIN group (p > 0.05). The detailed data of the percentages were illustrated in the representative flow cytometric figures and listed in Table 4. In addition, compared with the control group (0.13 ± 0.04), the TH17/Treg ratio in the UCC group (0.26 ± 0.11) and the CIN group (0.25 ± 0.12) were also significantly higher (p < 0.05). Taken together, these data suggest that in cervical cancer patients the levels Treg cells and Th17and the ratio of TH17/Treg were significantly changed.

**Table 4:** The proportions of Treg cells and Th17 cells of the three groups

**Percentages (%) of cells**	**UCC group**	**CIN group**	**Control group**
CD4^+^CD25^+^Treg	13.31 ± 2.02	12.75 ± 2.61	6.99 ± 1.62
CD4^+^CD25^+^ CD127^-^Treg	6.43 ± 1.04a	6.13 ± 1.23a	3.78 ± 0.70
CD8^+^CD25^+^CD127^-^Treg	3.01 ± 1.21	2.66 ± 1.45	1.73 ± 1.03
CD4^+^CD25^+^Foxp3^+^	6.81 ± 1.03	6.35 ± 1.75	2.81 ± 1.41
CD4^+^CD25^-^Foxp3^+^	3.52 ± 1.45	3.15 ± 1.38	1.53 ± 0.87
CD8^+^CD25^+^Foxp3^+^ Treg	0.81 ± 0.19	0.74 ± 0.23	0.27 ± 0.16
CD4^+^IL17^+^Th17	3.54 ± 1.44	3.29 ± 1.45	0.96 ± 0.54

Note: The quantitative results of flow cytometry analysis in Figure 2 were shown in this Table.

Expression levels of TGF-β, IFN-γ, IL-6, IL-10, IL-17 and IL-23 are elevated in cervical cancer patients and CIN patients.

**Figure 4:**
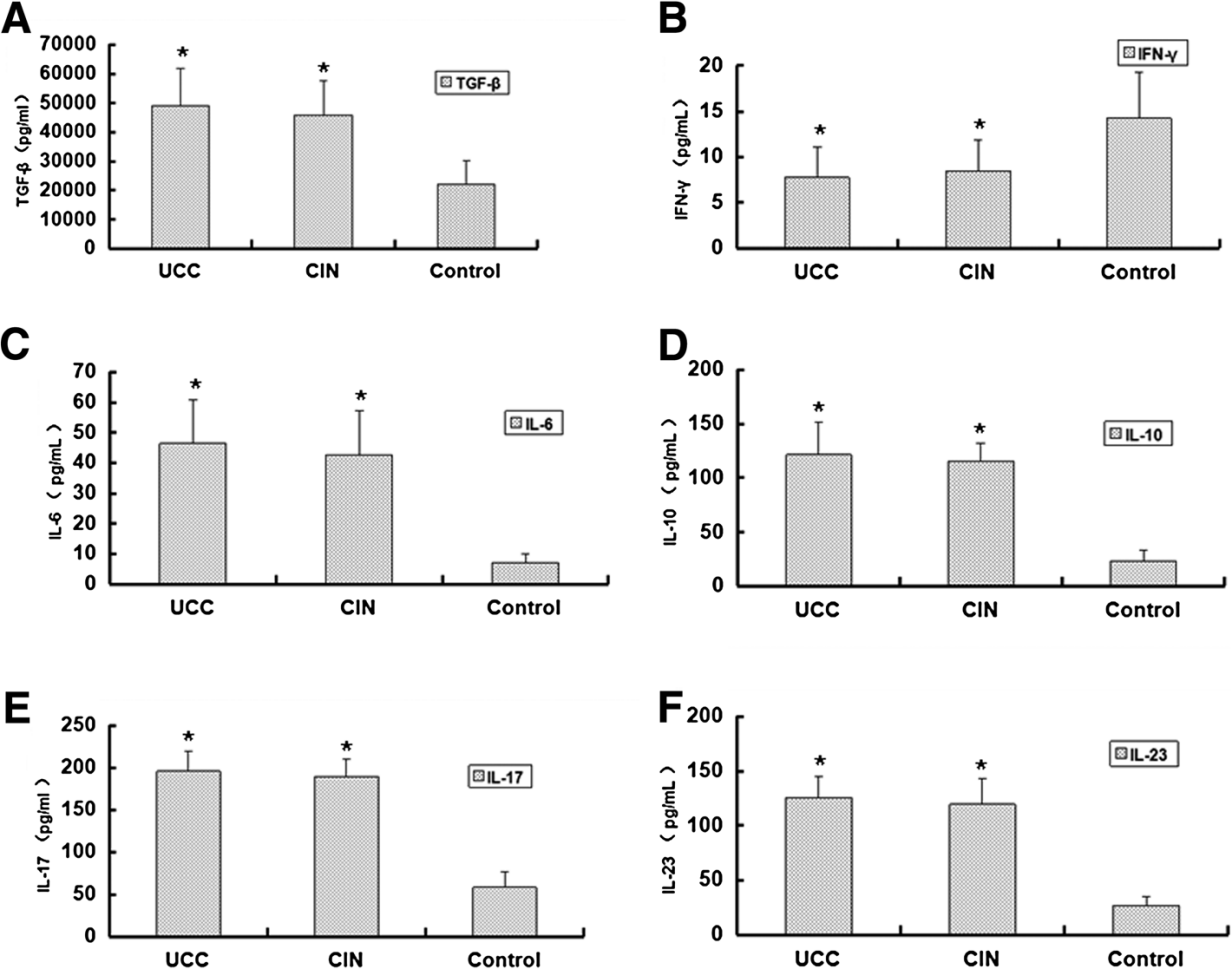
**Detection of related cytokines.** Peripheral blood samples were collected from 65 cases of cervical cancer patients, 42 cases of cervical CIN patients and 40 cases of healthy people. ELISA assay was performed to detect the expression levels of related cytokines in the serum of the three groups. (**A**) levels of TGF-β; (**B**) levels of IFN-γ; (**C**) levels of IL-6; (**D**) levels of IL-10; (**E**) levels of IL-17; (**F**) levels of IL-23. Each experiment was performed three times. Levels of cytokines in the UCC group and the cervical group were compared with those in the healthy control group. *p < 0.05.

ELISA assay was performed to measure the expression levels of the cytokines (including TGF-β, IFN-γ, IL-6, IL-10, IL-17 and IL-23) in the serum and the quantitative results were shown in Figure 4. The levels of TGF-β in the UCC group, the CIN group and the healthy control group were 49000 ± 12910 pg/mL, 46000 ± 11480 pg/mL and 22000 ± 8140 pg/mL, respectively (Figure 4A). The levels of IFN-γ in the UCC group, the CIN group and the healthy control group were 7.75 ± 3.32 pg/mL, 8.47 ± 3.35 pg/mL and 14.25 ± 4.95 pg/mL, respectively (Figure 4B). The levels of IL-6 in the UCC group, the CIN group and the healthy control group were 46.38 ± 14.38 pg/mL, 42.52 ± 14.78 pg/mL and 6.87 ± 3.13 pg/mL, respectively (Figure 4C). The levels of IL-10 in the UCC group, the CIN group and the healthy control group were 121.28 ± 30.09 pg/mL, 115.21 ± 16.74 pg/mL and 23.31 ± 9.79 pg/mL, respectively (Figure 4D). The levels of IL-17 in the UCC group, the CIN group and the healthy control group were 195.73 ± 23.48 pg/mL,189.74 ± 20.82 pg/mL and 58.37 ± 18.27 pg/mL, respectively (Figure 4E). The levels of IL-23 in the UCC group, the CIN group and the healthy control group were 125.55 ± 19.08 pg/mL, 119.43 ± 23.79 pg/mL and 26.67 ± 8.17 pg/mL, respectively (Figure 4F). Statistically, compared with the healthy control group, the expression levels of these cytokines in the UCC group and the CIN group were significantly higher (p < 0.05). Meanwhile, there was no significant difference between the UCC group and the CIN group (p > 0.05). Collectively, the expression levels of TGF-β, IFN-γ, IL-6, IL-10, IL-17 and IL-23 were up-regulated in cervical cancer patients. And these results suggest that immune response in cervical cancer patients was suppressed.

### Correlation analysis

**Figure 5:**
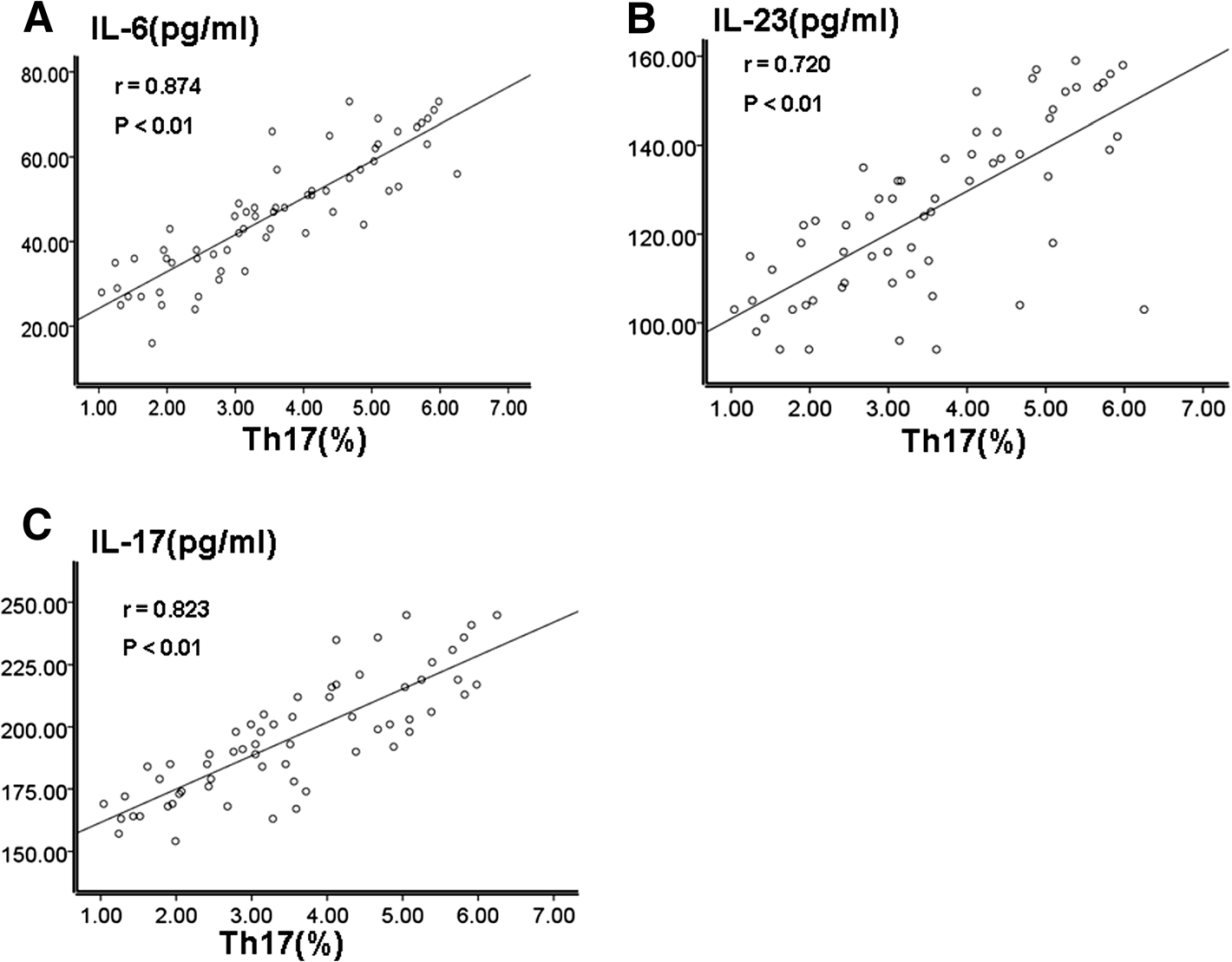
**Correlation analysis of levels of Th17 cells and levels of IL-6, IL-23 and IL-17 in the UCC group.** Correlation assay was conducted using the Spearman rank correlation test. (**A**) Correlation analysis between Th17 and IL-6; (**B**) Correlation analysis between Th17 and IL-23; (**C**) Correlation analysis between Th17 and IL-17.

**Figure 6:**
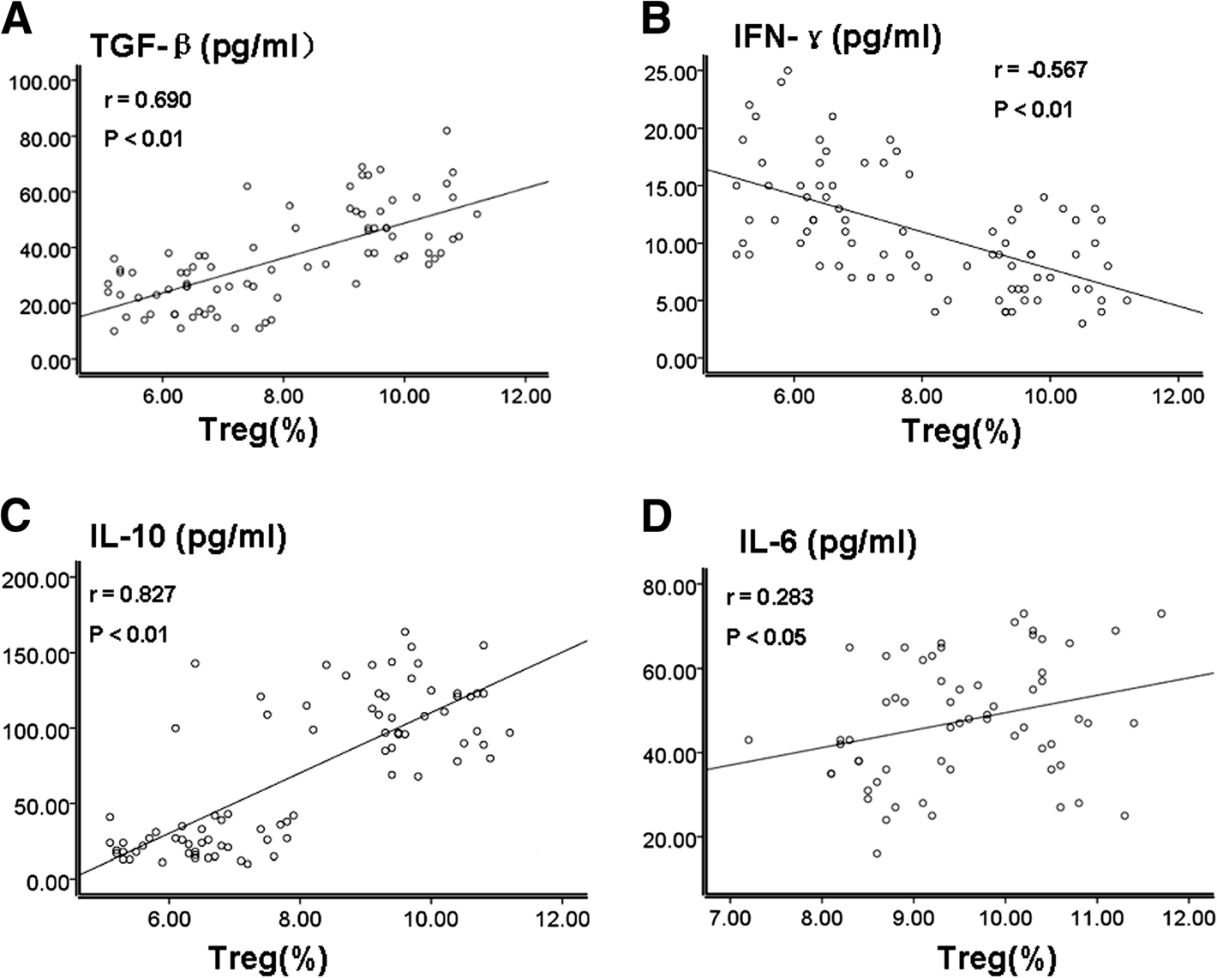
**Correlation analysis of levels of Treg cells and levels of TGF-β, IFN-γ, IL-10 and IL-6 in the UCC group.** Correlation analysis was performed by the Spearman rank correlation test. (**A**) Correlation analysis between Treg and TGF-β; (**B**) Correlation analysis between Treg and IFN-γ; (**C**) Correlation analysis between Treg and IL-10; (**D**) Correlation analysis between Treg and IL-6.

As mentioned above, percentages of Th17 cells and Treg cells were increased in cervical cancer patients. So were the levels of TGF-β, IFN-γ, IL-6, IL-10, IL-17 and IL-23 cytokines. In order to check whether these two elevations were correlated to each other, we did the correlation analysis in the UCC group. As shown in Figure 5, there was a positive correlation between percentages of Th17 cells and levels of IL-6, IL-23 and IL-17 cytokines (r = 0.874, p < 0.01, Figure 5A; r = 0.720, p < 0.01, Figure 5B; r = 0.823, p < 0.01, Figure 5C). Similarly, as shown in Figure 6A, C and D, there was a positive correlation between percentages of Treg cells and levels of TGF-β, IL-10 and IL-6 cytokines (r = 0.874, p < 0.01, Figure 6A; r = 0.720, p < 0.01, Figure 6C; r = 0.823, p < 0.01). On the contrary, there was a negative correlation between percentages of Treg cells and levels of IFN-γ cytokine (r = −0.567, p < 0.01, Figure 6B).

## Discussion

In our study, by studying the Treg/ Th17 balance and the related cytokines, we systematically investigated the cervical tumor immunology of Uygur women. The samples consisted of 65 cervical cancer patients, 42 cervical CIN patients and 40 healthy volunteers. The PBMCs were collected and stained with different cell surface markers before cytometric analysis. Levels of related cytokines were detected by ELISA kits. We hope that our findings would provide further experimental data for cervical tumor immunology.

Firstly, we analyzed the total percentages of peripheral CD3^+^ T cells, CD4^+^ T cells and CD8^+^ T cells. Our results showed that there were no significant differences in the total percentages of peripheral CD3^+^ T cells, CD4^+^ T cells and CD8^+^ T cells, and the ratio of CD4^+^/CD8^+^ (p > 0.05). These results showed that the T cell subsets of CD4^+^ and CD8^+^ cells in cancer patients were not lower than those in healthy individuals.

Then we detected the percentages of Treg cells. Treg cells are a kind of suppressor immune cells and play important roles in immune tolerance and homeostasis. Studies have shown that percentages of Treg cells increased in advanced tumor patients. This increase of Treg cells was correlated to the decrease of mortality. Dannull J et al. [10] depleted Treg cells by anti-CD25 antibody in tumor bearing mice. They found that there were effective anti-tumor immune responses in these mice. These results indicate a suppressive role of Treg cells in anti-tumor immune response. In addition, Liu et al. [11] reported that CD127 was one of the surface markers of Treg cells. In our study, we found that the percentages of CD4^+^CD25^+^CD127^lo/-^ Treg in CD4^+^ T cells in the UCC group, the CIN group and the control group were (6.43 ± 1.04)%, (6.13 ± 1.23)% and (3.78 ± 0.70)%, respectively. Compared with the control group, the percentages of CD4^+^CD25^+^CD127^lo/-^ Treg in the UCC group and the CIN group were significantly higher (p < 0.05). There was no significant difference between the UCC group and the CIN group (p > 0.05). Thus we speculate that CD4^+^CD25^+^CD127^lo/-^ Treg cells may play an important role in the development of cervical cancer. In cervical cancer patients, Treg cell-mediated immune tolerance may be closely related to tumor growth, and may be directly involved in tumorigenesis. Also there was a slight increase of the CD4^+^CD25^+^CD127^lo/-^ Treg cells in the UCC group compared with those in the CIN group. This data further suggest that with the progression of cervical cancer, the number of CD4^+^CD25^+^CD127^lo/-^ Treg cells was gradually increased. This increase further suppressed the immune response and weakened the anti-tumor response, leading to the growth and metastasis of the tumor.

On the other hand, Ike-moto et al. [12] reported that percentages of CD4^+^Foxp3^+^Treg cells in the peripheral blood of pancreatic cancer patients were significantly higher. Similarly, CD4^+^Foxp3^+^Treg cells were also higher in prostate cancer bearing mice and glioma tumor bearing mice [13,14]. These results suggest that CD4^+^Foxp3^+^Treg cell levels were closely related with tumors. Naive CD4^+^ T cells can be transformed into CD4^+^ CD25^+^ T cells by introducing Foxp3. CD4^+^ CD25^+^ T cells lack of Foxp3 did not induce the suppressor function [15]. Our study showed that the CD4^+^Foxp3^+^ Treg cells in the UCC group and the healthy control group were (3.52 ± 1.45)% and (1.53 ± 0.87)%. And the CD4^+^CD25^+^Foxp3^+^ Treg in the UCC group and the healthy control group were (6.81 ± 1.03)% and (2.81 ± 1.41)%. Statistically, the CD4^+^Foxp3^+^ Treg cells and CD4^+^CD25^+^Foxp3^+^ Treg cells in UCC group were significantly higher than the healthy control group (p <0.05). The same significant difference was also found between the cervical CIN group and the healthy control group. Meanwhile there was no significant difference between the UCC group and the cervical CIN group. However the percentages of Foxp3^+^ Treg cells in the cervical CIN group were slightly higher than the UCC group, indicating that with the progression of cancer, percentages of Foxp3^+^ Treg cells gradually increased. It is known that Foxp3^+^ Treg cells could inhibit anti-tumor function of CD8^+^ T cells in vitro. Hence it is reasonable to speculate that up-regulation of Foxp3^+^ Treg cells in cervical CIN and cervical cancer patients will lead to weakened immune surveillance function. Therefore Foxp3^+^ Treg cells up-regulation may be the underlying mechanisms of cervical cancer genesis and development.

Studies found that in prostate cancer [16,17] and colorectal cancer [18] patients, the numbers CD8^+^CD25^+^Foxp3^+^ T cells in the peripheral blood of patients and the local regions of cancer increased. CD8^+^CD25^+^CD127^lo/-^ Treg cells have a similar function. In this study, the percentages of CD8^+^CD25^+^Foxp3^+^ T cells in the UCC group and the control group were (0.81 ± 0.19)% and (0.27 ± 0.16)%. The percentages of CD8^+^CD25^+^CD127^lo/-^ Treg cells in the UCC group and the control group were (3.01 ± 1.21)% and (1.73 ± 1.03)%, respectively. Statistically, the CD8^+^CD25^+^Foxp3^+^ T cells and CD8^+^CD25^+^CD127^lo/-^ Treg cells in UCC group were significantly higher than the control group (p <0.05). Similarly the statistically significant difference was also found between the patients of the CIN group and individuals of the control group (p < 0.05). No statistically significant difference was found between the UCC group and the cervical CIN group (p > 0.05). However, with the development of the disease, the levels of these two cells in the UCC group and the cervical CIN group gradually increased. CD8^+^CD25^+^ Treg cells are newly discovered regulatory T cells and have immunosuppressive effects, resulting in an immunocompromised state of patient. In our study, we found that CD8^+^CD25^+^ Treg cells were up-regulated in cervical cancer patients, suggesting that CD8^+^CD25^+^ Treg cells may play a role in the inducement and maintenance of immune tolerance in cervical cancer patients. Treatment targeting CD8^+^CD25^+^ Treg cells may be a promising measure in the future immunotherapy for cervical cancer.

Several studies have shown that Th17 cells and Treg cells were closely related with malignant tumors such as gastric cancer, ovarian cancer and colon cancer [19-22]. We found similar results in this study. We found that the percentages of Th17 cells in the UCC group (3.54% ± 1.44%) were significantly higher than those in the control group (0.96% ± 0.54%) (p < 0.05). Also statistically significant difference was also found between the cervical CIN group and the control group. At the same time, there was no significant difference between the UCC group and the cervical CIN group. Yet the percentages of Th17 cells increased gradually with the progression of cancer. So was the ratio of TH17/Treg. Beriou et al. [23] found that in the presence of strong TCR signaling and APC, Treg cells could secrete IL-17. Furthermore, in the tumor microenvironment producing IL-17, Treg cells could be transformed into Th17 cells, which further amplified the inflammatory response. The transformation between the Treg cells and Th17 cells may determine the growth and regression of tumor.

Cytokines are important mediators of immune responses, including immune responses in tumor environment. During tumorigenesis, the roles of some cytokines are ambivalent. For example, TGF-β could promote tumor growth through diminishing the production of IFN-γ and stimulating Treg cells. On the other hand, cytokines of TGF-β, IL-6 and IL-23 could promote the development of Th17 cells, which could enhance the immune response in the tumor environment and inhibit tumor growth. As mentioned above, we detected the changes in the percentages of Th17 and Treg cells in the peripheral blood of cervical cancer patients. To further investigate the role of cytokines in cervical cancer bearing patients, we measured the related cytokines of Th17 and Treg cells in the peripheral blood of cervical cancer patients. The following cytokines of TGF-β, IL-6, IL-10, IL-17, IL-23 and IFN-γ were measured. Also, the correlation of these cytokines with Th17 and Treg cells was analyzed in the UCC group.

It is known that IFN-γ is a tumor-killing cytokine produced by CTL and NK cells. CTL and NK cells are the primary tumor immunity cells in the body. In this study, the expression levels of IFN-γ were also analyzed. We found that IFN-γ levels in patients with cervical cancer and cervical CIN were significantly lower than those in the control group. Also IFN-γ levels were negatively correlated with CD4^+^CD25^+^CD127^lo/-^ Treg cell levels. This data suggest that Treg cells may suppress CTL and NK cells to produce IFN-γ cytokine.

Th17 cells could secrete IL-17, which could promote tumor angiogenesis and further promote tumorigenesis and development. One in vivo study transfected NSCLC cells with IL-17 expressing plasmids and then transplanted these cells into SCID mice. They found that tumors formed by these cells growed much faster [24]. This result further illustrates that IL-17’s role of promoting angiogenesis was through increasing the chemotaxis of vascular endothelial. Our results were consistent with this study. We found that levels of TGF-β, IL-6, IL-10, IL −17 and IL-23 in patients with cervical cancer and cervical CIN were significantly higher than those in the control group. Furthermore there was a positive correlation between the percentages of Th17 cells (r = 0.874, p < 0.01; r = 0.720, p < 0.01; r = 0.823, p < 0.01) and the levels of IL-6, IL-23 and IL-17. And the percentages of Treg cells was positively correlated with levels of TGF-β, IL-10 and IL-6 (r = 0.690, p < 0.01; r = 0.827, p < 0.01; r = 0.283, p < 0.05). These data suggest that cytokines of TGF-β, IL-6, IL-17 and IL-23 promoted the imbalance of Th17/Treg ratio. The increase of these cytokines may reduce the body’s immune function and promote tumor development by affecting the Th17/Treg ratio. Thus these changes might be the possible reasons that promoted the development of cervical cancer.

## Conclusions

In summary, our study concluded that elevations of CD4^+^CD25^+^CD127^lo/-^Treg, Foxp3^+^ Treg, CD8^+^ Treg and Th17 cells exsit in peripheral blood of Uygur patients with cervical cancer. And these changes may be one of the reasons for low immune function. And one of the possible immune mechanisms for Uygur cervical cancer was TH17/Treg imbalance. In the future, enhancing the body’s immune surveillance function could be done through reducing the levels of CD4^+^CD25^+^CD127^lo/-^Treg, Foxp3^+^ Treg, CD8^+^ Treg and Th17 cells or reversing their phenotypes, or correcting the imbalance of Th17/Treg ratio. And this may provide a new therapeutic approach for improving the anti-tumor immunity.

## Abbreviations

UCC group: Uygur cervical cancer group; CIN group: Cervical intraepithelial neoplasia group.

## Competing interests

The authors declare that they have no competing interests.

## Authors’ contributions

ZC and JD did experiments and prepared the manuscript. NP, RD, and WM did experiments. YuZ, YZ, and CM analyzed the data. YD designed the study. All authors read and approved the final manuscript.
